# SAV4189, a MarR-Family Regulator in *Streptomyces avermitilis*, Activates Avermectin Biosynthesis

**DOI:** 10.3389/fmicb.2018.01358

**Published:** 2018-06-26

**Authors:** Jia Guo, Xuan Zhang, Xiaorui Lu, Wenshuai Liu, Zhi Chen, Jilun Li, Linhong Deng, Ying Wen

**Affiliations:** ^1^State Key Laboratory of Agrobiotechnology, College of Biological Sciences, China Agricultural University, Beijing, China; ^2^Institute of Biomedical Engineering and Health Sciences, Changzhou University, Changzhou, China; ^3^Key Laboratory of Carbohydrate Chemistry and Biotechnology, Ministry of Education, School of Biotechnology, Jiangnan University, Wuxi, China

**Keywords:** *Streptomyces avermitilis*, avermectins, MarR-family regulator, SAV4189, *sav_4190*

## Abstract

The bacterial species *Streptomyces avermitilis* is an important industrial producer of avermectins, which are widely utilized as effective anthelmintic and insecticidal drugs. We used gene deletion, complementation, and overexpression experiments to identify SAV4189, a MarR-family transcriptional regulator (MFR) in this species, as an activator of avermectin biosynthesis. SAV4189 indirectly stimulated avermectin production by altering expression of cluster-situated activator gene *aveR*, and directly repressed the transcription of its own gene (*sav_4189*) and adjacent cotranscribed gene *sav_4190* (which encodes an unknown transmembrane efflux protein). A consensus 13-bp palindromic sequence, 5′-TTGCCYKHRSCAA-3′ (Y = T/C; K = T/G; H = A/C/T; R = A/G; S = C/G), was found within the SAV4189-binding sites of its own promoter region, and shown to be essential for binding. The SAV4189 regulon was thus predicted based on bioinformatic analysis. Night new identified SAV4189 targets are involved in transcriptional regulation, primary metabolism, secondary metabolism, and stress response, reflecting a pleiotropic role of SAV4189. *sav_4190*, the important target gene of SAV4189, exerted a negative effect on avermectin production. *sav_4189* overexpression and *sav_4190* deletion in *S. avermitilis* wild-type and industrial strains significantly increased avermectin production. SAV4189 homologs are widespread in other *Streptomyces* species. *sav_4189* overexpression in the model species *S. coelicolor* also enhanced antibiotic production. The strategy of increasing yield of important antibiotics by engineering of SAV4189 homologs and target gene may potentially be extended to other industrial *Streptomyces* species. In addition, SAV4189 bound and responded to exogenous antibiotics hygromycin B and thiostrepton to modulate its DNA-binding activity and transcription of target genes. SAV4189 is the first reported exogenous antibiotic receptor among *Streptomyces* MFRs.

## Introduction

*Streptomyces* are filamentous soil bacteria characterized by complex life cycle and production of numerous antibiotics (van Wezel and McDowall, 2011). Antibiotic biosynthetic gene clusters (BGCs) are tightly controlled by cluster-situated regulators (CSRs) and higher-level pleiotropic regulators that respond to environmental and physiological signals (van Wezel and McDowall, 2011; Liu G. et al., 2013; Urem et al., 2016). However, the signals and their transduction systems that control expression of specific BGCs are poorly understood, because genome sequencing has shown that *Streptomyces* species contain numerous BGCs, some of them are not expressed under laboratory conditions and called cryptic BCGs. Activation of cryptic BGCs provides an important source for discovery of new compounds (Rutledge and Challis, 2015). Identification and manipulation of transcriptional regulators involved in antibiotic biosynthesis will facilitate discovery of new antibiotics and construction of antibiotic-overproducing strains for industrial applications.

At least 20 families of transcriptional regulators are found in *Streptomyces*, including TetR, LuxR, LysR, AraC, GntR, SARP, ROK, MerR, and MarR (multiple antibiotic resistance regulator). MarR-family transcriptional regulators (MFRs) comprise more than 19,000 members and are widespread in bacteria and archaea. MFRs control a variety of cellular processes, including multidrug resistance, pathogenicity, stress responses, metabolic pathways, and degradation of aromatic compounds (Perera and Grove, 2010; Romero-Rodríguez et al., 2015). They have characteristic winged helix-turn-helix (wHTH) DNA-binding domains and typically act as homodimeric transcriptional repressors (sometimes as activators, or both) by binding to palindromic sequences within target promoter regions. DNA-binding affinity of MFRs is reduced by conformational changes in response to small-molecule ligands, resulting in blocking of transcriptional regulation, but the natural ligands are often unknown (Perera and Grove, 2010; Grove, 2013). MFRs are the fourth most abundant family of transcriptional regulators in *Streptomyces* (average 50 per genome), but only a few members have been characterized in this genus, including *Streptomyces coelicolor* OhrR (Oh et al., 2007), TamR (Huang and Grove, 2013), PecS (Huang et al., 2013), and PcaV (Davis et al., 2013), *S. exfoliatus* PenR, *S. treptomyces arenae* PntR (Zhu et al., 2013), and *S. roseosporus* DptR3 (Zhang et al., 2015).

Avermectins produced by *S. avermitilis* are economically important, potent anthelmintic and insecticidal drugs widely utilized in human medicine, animal husbandry, and agriculture (Burg et al., 1979; Egerton et al., 1979). There are eight avermectin components (A1a, A1b, A2a, A2b, B1a, B1b, B2a, B2b), among which B1a has the highest insecticidal activity. The regulatory mechanisms of avermectin biosynthesis have been intensively studied. The 82-kb *ave* gene cluster contains only one CSR gene, *aveR*, which encodes a LAL family activator required for expression of *ave* biosynthetic genes (Kitani et al., 2009; Guo et al., 2010). Our recent studies have shown that PhoP (response regulator of two-component system PhoR/PhoP) (Yang et al., 2015), AvaR1 (avenolide autoregulator receptor) (Zhu et al., 2017), and AvaR2 (homolog of pseudo γ-butyrolactone receptor) (Zhu et al., 2016) are direct repressors of *aveR*, and that SA742 (AraC-family regulator) directly represses several *ave* structural genes, rather than *aveR* (Sun et al., 2016). Transcriptional regulators shown to indirectly control avermectin production include TetR-family regulators AveI (SAV4110) (Chen et al., 2008), SAV7471 (Liu Y. et al., 2013), SAV151 (He et al., 2014), SAV576 (Guo et al., 2013), SAV577 (Guo et al., 2014), and AveT (SAV3619) (Liu et al., 2015), ECF-σ^25^ (Luo et al., 2014), and alternative σ factor σ^8^ (Sun et al., 2017). Despite these findings, our knowledge of the complex regulatory network underlying avermectin biosynthesis is still limited and fragmentary, and presents an obstacle to rational design of avermectin high-producing strains through genetic manipulation.

Forty-two MFRs are encoded by the *S. avermitilis* genome, but none of them have been characterized. In a search for new regulators that affect avermectin biosynthesis, we previously applied a *S. avermitilis* whole-genome chip to compare transcriptomes of wild-type strain and avermectin high-producer 76–02-e (Guo et al., 2013). We observed that transcription level of *sav_4189*, which encodes a MFR, was greatly upregulated in 76–02-e, suggesting that SAV4189 is involved in control of avermectin biosynthesis. In the present study, we examined the regulatory role of SAV4189, and found that it functions as an indirect activator of avermectin production. An effective strategy was developed for enhancing avermectin yield in industrial strains through overexpression of SAV4189 and deletion of its target gene, *sav_4190*. Exogenous antibiotics hygromycin B (HygB) and thiostrepton (Thi), produced by other *Streptomyces* species, released SAV4189 from its target genes. Thus, SAV4189 appears to mediate interspecies communication in *Streptomyces* by responding to antibiotic signals.

## Materials and Methods

### Strains, Plasmids, and Growth Conditions

Plasmids and bacterial strains used/constructed in this work are summarized in **Table 1**, and primers are listed in Supplementary Table S1. Culture conditions for *S. avermitilis* and *Escherichia coli* were as described previously (Liu et al., 2015). YMS (Ikeda et al., 1988), SFM (Kieser et al., 2000), MM (Kieser et al., 2000), and RM14 (MacNeil and Klapko, 1987) agar were used for observation of *S. avermitilis* phenotype. *S. coelicolor* M145 was grown at 28°C and used as a heterogeneous host for overexpression of SAV4189. YBP agar (Ou et al., 2009) was used for observation of *S. coelicolor* phenotype. For routine avermectin production, insoluble fermentation medium FM-I was used (Chen et al., 2007). For growth analysis, soluble fermentation medium FM-II was used (Guo et al., 2010).

**Table 1:** Strains and plasmids used in this study.

Strain or plasmid	Description	Source
*S. avermitilis*		
ATCC31267	Wild-type (WT) strain	Laboratory stock
A-144	Industrial strain	Qilu Pharmaceutical
D4189	*sav_4189* deletion mutant	This study
C4189	*sav_4189* complemented strain	This study
O4189	*sav_4189* overexpression strain	This study
O4189/A-144	*sav_4189* overexpression strain based on A-144	This study
D4190	*sav_4190* deletion mutant	This study
D4190/A-144	*sav_4190* deletion mutant based on A-144	This study
*S. coelicolor*		
M145	WT strain	Laboratory stock
O4189/M145	*sav_4189* overexpression strain based on M145	This study
*E. coli*		
JM109	Routine cloning host	Laboratory stock
ET12567	Methylation-deficient strain	MacNeil and Klapko, 1987
BL21(DE)	Host for His_6_-SAV4189 overexpression	Novagen
Plasmids		
pKC1139	Multiple-copy, temperature-sensitive *E. coli*–*Streptomyces* shuttle plasmid	Bierman et al., 1992
pSET152	Integrative *E. coli*–*Streptomyces* shuttle plasmid	Bierman et al., 1992
pET-28a (+)	Plasmid for His_6_-tagged protein overexpression in *E. coli*	Novagen
pMD18-T	TA cloning vector	TaKaRa
pJL117	Plasmid carrying *Streptomyces* strong constitutive promoter *ermE^∗^p*	Li et al., 2010
pD4189	pKC1139 derivative for *sav_4189* deletion	This study
pKC1139-4189	pKC1139 derivative for *sav_4189* overexpression	This study
pSET152-4189	pSET152 derivative for *sav_4189* complementation	This study
pD4190	pKC1139 derivative for *sav_4190* deletion	This study
pET28-4189	pET-28a (+) derivative for His_6_-SAV4189 overexpression	This study


### Gene Disruption, Complementation, and Overexpression

To construct a *sav_4189* deletion mutant, two flanking fragments of *sav_4189* were prepared by PCR using genomic DNA of wild-type (WT) strain ATCC31267 as template. A 511-bp 5′ flanking region (from positions -314 to +197 relative to the *sav_4189* start codon) was amplified with primers ZX141 and ZX142, and a 522-bp 3′ flanking region (from positions +502 to +1023) was amplified with primers ZX143 and ZX144. The two fragments were fused by PCR using primers ZX141 and ZX144, and ligated into pKC1139 to generate *sav_4189* deletion vector pD4189, which was transformed into WT protoplasts. *sav_4189*-deleted mutant D4189 was selected by the method described previously (Zhao et al., 2007), confirmed by PCR analysis using primers ZX235, ZX236, ZX237, and ZX238 (Supplementary Figure S1A), and subjected to DNA sequencing. When using primers ZX235/ZX236 (flanking the exchange regions), a 1.4-kb band was observed in D4189, whereas a 1.7-kb band was produced from WT genomic DNA. When using primers ZX237/ZX238 (located within the deletion region), only WT produced a 0.4-kb PCR fragment as predicted (data not shown).

For complementation of D4189, a 607-bp DNA fragment carrying the *sav_4189* ORF was obtained by PCR with primers ZX149 and ZX150, and ligated simultaneously with a 264-bp fragment containing promoter *ermE^∗^p* from pJL117 into pSET152 to generate *sav_4189*-complemented vector pSET152-4189, which was then introduced into D4189 to obtain complemented strain C4189. The two fragments containing *sav_4189* ORF and *ermE^∗^p* were ligated simultaneously into pKC1139 to produce *sav_4189* overexpression vector pKC1139-4189, which was then introduced into *S. avermitilis* WT and industrial strain A-144 to obtain *sav_4189* overexpression strains O4189 and O4189/A-144, respectively. pKC1139-4189 was transformed into *S. coelicolor* M145 to obtain O4189/M145.

To construct a *sav_4190* deletion mutant, a 585-bp 5′ flanking region (from positions -481 to +104 relative to the *sav_4190* start codon), and a 575-bp 3′ flanking region (from positions +2446 to +3020) were amplified with primer pairs ZX177/ZX178 and ZX179/ZX180, respectively. The two fragments were ligated into pKC1139 to obtain *sav_4190* deletion vector pD4190, which was transformed into WT protoplasts. The resulting *sav_4190*-deleted mutant D4190 was confirmed by PCR using ZX239, ZX240, ZX241, and ZX236 as primers (Supplementary Figure S1B). When using primers ZX239/ZX240 (flanking the exchange regions), a 1.5-kb band appeared in D4190, whereas a 3.8-kb band was produced from WT. When using primers ZX241/ZX236 (located within the deletion region), only WT produced a 0.5-kb PCR fragment as predicted (data not shown). To delete *sav_4190* in A-144, the vector pD4190 was transformed into A-144 protoplasts. The expected mutant, termed D4190/A-144, was isolated by selection of the D4190 mutant and confirmed by PCR using the same primer pairs.

### Analysis of Avermectin Production

Avermectins were extracted by methanol from *S. avermitilis* fermentation cultures, and analyzed by HPLC as described previously (Chen et al., 2007).

### Scanning Electron Microscopy (SEM)

Spores and mycelia of *S. avermitilis* WT and D4189 strains grown on SFM agar for 4 days were observed by SEM. Specimens were prepared and examined as described previously (Sun et al., 2016).

### Quantitative Real-Time RT-PCR (qRT-PCR) Analysis

Total RNAs were isolated at various times from *S. avermitilis* cultures grown in FM-I, and qRT-PCR was conducted as described previously (Luo et al., 2014) to determine transcription levels of tested genes using primers listed in Supplementary Table S1. *hrdB* gene (*sav_2444*, encoding the principal sigma factor) was used as internal control for normalization of transcription levels. Gene expression was determined in triplicate and repeated for three independent samples.

### Overexpression and Purification of His_6_- SAV4189

The 516-bp *sav_4189* coding region (171 amino acids) was amplified by PCR with primers ZX205 and ZX206. The PCR fragment was digested with *Nde*I/*Eco*RI and inserted into expression vector pET-28a (+) to generate pET28-4189, which was verified by DNA sequencing. pET28-4189 was transformed into *E. coli* BL21 (DE3), and recombinant His_6_-SAV4189 protein overexpression was induced by 0.2 mM IPTG for 8 h at 16°C. Cells were collected, washed, and disrupted in lysis buffer (50 mM NaH_2_PO_4_, 300 mM NaCl, 10 mM imidazole, pH 8.0) by sonication on ice. The preparation was centrifuged, and the supernatant containing soluble His_6_-SAV4189 was loaded onto a Ni^2+^-NTA column (Qiagen). After extensive washing with wash buffer (50 mM NaH_2_PO_4_, 300 mM NaCl, 50 mM imidazole, pH 8.0), the His_6_-SAV4189 protein was specifically eluted from the resin with elution buffer (50 mM NaH_2_PO_4_, 300 mM NaCl, 200 mM imidazole, pH 8.0). Concentrations of purified proteins were determined by Bradford assay, and proteins were stored at -80°C.

### Electrophoretic Mobility Shift Assays (EMSAs)

Electrophoretic mobility shift assays were performed as described previously (Luo et al., 2014). DNA probes harboring promoter regions of tested genes were obtained by PCR using corresponding primers (Supplementary Table S1), labeled with non-radioactive digoxigenin (DIG) at the 3′ end, and incubated individually with various amounts of His_6_-SAV4189 in binding reaction. To verify specificity of SAV4189-probe *4189p* interaction, a ∼100-fold excess of unlabeled specific probe *4189p* or non-specific probe *hrdB* was added to the reaction mixture.

For EMSAs with antibiotics, avermectin B1 (AveB1), oligomycin (Oli), and thiostrepton (Thi) were dissolved in DMSO, while hygromycin B (HygB), apramycin (Apr), and kanamycin (Kan) were dissolved in deionized water. Dissolved antibiotics were added at various final concentrations, and solvent control DMSO was added at 8 μl in the reaction mixture.

### Determination of Transcriptional Start Site (TSS)

5′ rapid amplification of cDNA ends (5′ RACE) analysis was performed to identify the *sav_4189* TSS using a 5′/3′ RACE kit (Roche). Total RNA (2 μg) prepared from 84-h culture of WT grown on YMS was used for reverse transcription with 20 pmol of gene-specific primer 4189SP1. An oligo(dA) tail was added to the 3′ end of purified cDNA using terminal deoxynucleotidyltransferase, followed by PCR amplification of the tailed cDNA using specific nested primer 4189SP2 and oligo(dT) anchor primer. The resulting PCR product (diluted 1000-fold) was amplified in an additional round of PCR with nested primer 4189SP3 and an anchor primer, to yield a single specific band. The purified final PCR product was inserted into pMD18-T vector (TaKaRa) for sequencing. The first nucleotide following oligo(dA) sequence was determined as the complementary base of *sav_4189* TSS.

### DNase I Footprinting Assay

A non-radiochemical capillary electrophoresis technique (Zianni et al., 2006) was used for DNase I footprinting with some modifications (Guo et al., 2013). To investigate binding sites of SAV4189 on its own promoter region, a 511-bp 5′ FAM fluorescence-labeled DNA probe covering the entire *sav_4188*-*sav_4189* intergenic region was synthesized by PCR using primers FAM-89footS/89footAS, and purified. The probe (400 ng) and various concentrations of His_6_-SAV4189 were mixed in a 25-μl volume, and incubated for 30 min at 25°C. Following DNase I digestion, DNA samples were purified and subjected to capillary electrophoresis with 3730XL DNA analyzer (Applied Biosystems). Results were processed with GeneMarker v2.2.

## Results

### SAV4189 Is an Activator of Avermectin Production

**FIGURE 1:**
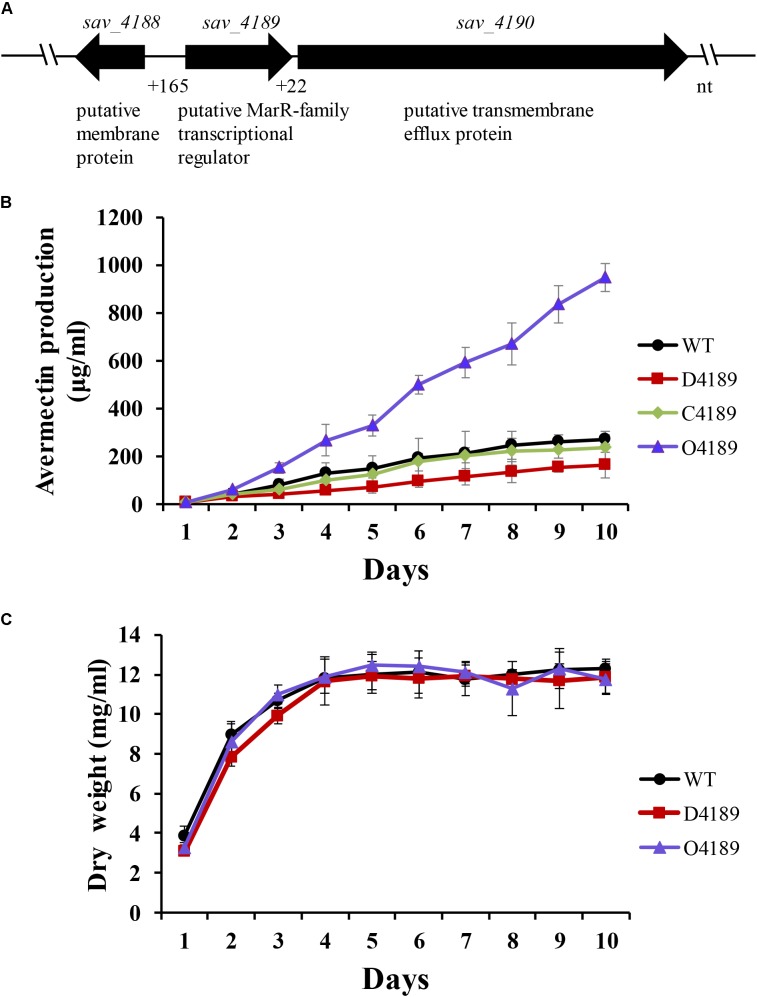
Effects of SAV4189 on avermectin production and growth in *Streptomyces avermitilis*. **(A)** Organization of *sav_4189* and its adjacent genes. **(B)** Avermectin yield in WT, *sav_4189* deletion mutant (D4189), complemented strain (C4189), and overexpression strain (O4189) cultured in FM-I. Avermectin yield contains total yield of all eight components A1a, A1b, A2a, A2b, B1a, B1b, B2a, and B2b. NS, not significant; ^∗∗^*P*< 0.01 (comparison with WT by Student’s *t*-test). **(C)** Growth curves of WT, D4189, and O4189 cultured in FM-II. In this and subsequent figures, error bars represent SD of three replicates.

The *sav_4189* gene contains 516 nucleotides (nt) and encodes a MFR of 171 amino acids (including a conserved wHTH DNA-binding motif homologous to MarR) whose function is unknown. Divergently transcribed gene *sav_4188*, located 165 nt upstream of *sav_4189*, encodes an unknown membrane protein. Convergently transcribed gene *sav_4190*, located downstream of *sav_4189*, encodes a putative transmembrane efflux protein (**Figure 1A**). The *sav_4189*-*sav_4190* intergenic region in only 22 nt long, suggesting that the two genes might be cotranscribed, and this was confirmed by RT-PCR (Supplementary Figure S2). BLAST analysis revealed that the genetic organization of *sav_4188*, *sav_4189* and *sav_4190* homologous genes is conserved among different *Streptomyces* species and SAV4189 homologs have high amino acid sequence identities (72–80%) (Supplementary Figure S3), reflecting the important biological function of this MFR in the genus.

To clarify the role of *sav_4189* in avermectin biosynthesis, we constructed *sav_4189* deletion mutant D4189, complemented strain C4189, and overexpression strain O4189, and compared their total avermectin yields (from 1 to 10 days culture in FM-I) with that of WT. HPLC analysis showed that avermectin yield in WT strain was not observable until day 2, then increased gradually from day 2 onward (**Figure 1B**). Relative to WT level, yields were lower for D4189, higher for O4189, and not significantly different for C4189. On the end of fermentation day 10, overexpression of *sav_4189* (strain O4189) increased avermectin yield by ∼2.5-fold, and deletion of *sav_4189* (strain D4189) led to a clear reduction (∼40%) in avermectin yield (**Figure 1B**). These findings indicate that SAV4189 plays an activator role in avermectin production.

To evaluate the effect of SAV4189 on cell growth, we measured biomass (dry cell weight) of WT, D4189, and O4189 cultured in soluble FM-II. Cell weights of the three strains were similar (**Figure 1C**), indicating that SAV4189 is not involved in regulation of cell growth, and that the changed avermectin yields of D4189 and O4189 did not result from alteration of cell growth. D4189 and O4189 grew normally on YMS, SFM, RM14, and MM plates (Supplementary Figure S4A). In SEM observations, degree of separation of aerial hyphae, spore size, and spore shape for D4189 were nearly identical as for WT (Supplementary Figure S4B), indicating that SAV4189 has no effect on morphological development.

### SAV4189 Activates *aveR* but Represses Its Own Gene and Adjacent Gene *sav_4190*

**FIGURE 2:**
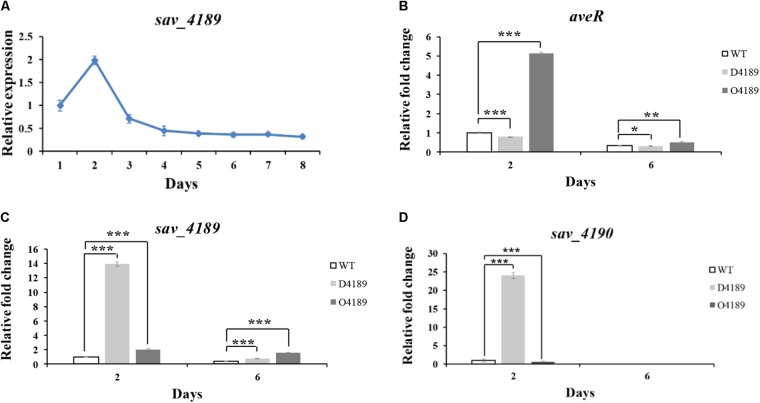
Transcriptional analysis of *aveR*, *sav_4189*, and *sav_4190* by qRT-PCR. **(A)** Transcriptional profile of *sav_4189* during the avermectin fermentation process in WT grown in FM-I. Value of *sav_4189* on day 1 was defined as 1. **(B)** *aveR*, **(C)** *sav_4189*, and **(D)** *sav_4190* transcription levels in WT, D4189, and O4189 grown in FM-I. Value of each gene was calculated relative to WT value on day 2, defined as 1. *sav_4189*: 91-bp transcript amplified from remainder *sav_4189* ORF in D4189 with primers ZX9 and ZX10. ^∗^*P*< 0.05, ^∗∗^*P*< 0.01, ^∗∗∗^*P*< 0.001.

To elucidate the relationship between SAV4189 and avermectin production, we examined the *sav_4189* transcription profile of WT grown in FM-I by qRT-PCR. *sav_4189* transcription level varied during the avermectin production process; it reached a maximum on day 2 when avermectin production was initiated, then declined sharply, and remained low from day 4 onward (**Figure 2A**). Thus, SAV4189 evidently exerts its regulatory function mainly during early fermentation stage.

To determine whether SAV4189 regulates avermectin production through CSR AveR, we performed qRT-PCR to examine *aveR* transcription levels of WT, D4189, and O4189 cultured in FM-I for 2 or 6 days. On both days, level relative to WT was reduced in D4189 and increased in O4189 (**Figure 2B**), consistently with avermectin yields. Notably, *aveR* transcription level was particularly high for O4189 on day 2. These findings indicate that SAV4189 regulates avermectin production by stimulating cluster-situated activator gene *aveR*, primarily during early fermentation stage.

A common regulatory mechanism of MFRs involves MarR protein regulating transcription of its own gene and a divergently oriented gene by binding to their intergenic region, which contains TSSs for both genes (Perera and Grove, 2010; Grove, 2013). *sav_4189* and *sav_4190* are cotranscribed. We therefore predicted that SAV4189 regulates expression of *sav_4189*-*sav_4190* operon and adjacent divergently transcribed gene *sav_4188*. We measured transcription levels of these three genes using the same RNA samples as for examining *aveR* expression. No *sav_4188* transcription was detectable in any of the three strains WT, D4189, and O4189, suggesting that this gene transcription was at very low level, or was not expressed under our fermentation conditions. We therefore did not investigate *sav_4188* in subsequent experiments. In O4189, *sav_4189* transcription level was higher than in WT, confirming *sav_4189* overexpression (**Figure 2C**). *sav_4190* transcription was slightly reduced on day 2, and undetectable on day 6 (**Figure 2D**). In D4189, transcription of both *sav_4189* and *sav_4190* was strongly upregulated on day 2. Transcription of *sav_4189* was slightly increased, and that of *sav_4190* was undetectable on day 6 (**Figures 2C,D**). These findings indicate that SAV4189 functions as a repressor of its own operon *sav_4189*-*sav_4190*, mainly during early fermentation stage.

### SAV4189 Binds Specifically to Its Own Gene Promoter Region

**FIGURE 3:**
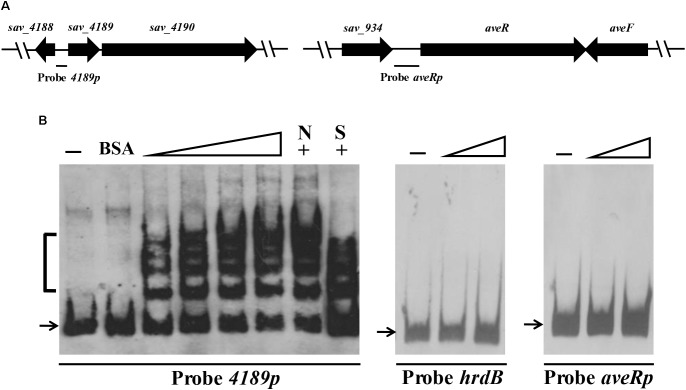
Electrophoretic mobility shift assays (EMSAs) of SAV4189 binding to its own promoter region. **(A)** Schematic diagram of probes used for EMSAs. Probe *4189p*: 148-bp DNA fragment, positions –145 to +3 relative to *sav_4189* translational start codon. Probe *aveRp*: 501-bp DNA fragment, positions –476 to +25 relative to *aveR* translational start codon. **(B)** Interaction of His_6_-SAV4189 with probes *4189p* and *aveRp*. Negative probe: *hrdB*, 118-bp DNA fragment within *hrdB* ORF. Negative protein control: 750 nM BSA. 0.15 nM labeled probe was added in each reaction. Concentrations of His_6_-SAV4189 for probes: for *4189p*, 12.5, 25, 50, and 62.5 nM; for *aveRp* and *hrdB*, 125 and 250 nM. 62.5 nM His_6_-SAV4189 was used for competition experiments (lanes +). Lanes –: EMSAs without His_6_-SAV4189. Lanes N and S: competition assays with ∼100-fold excess of unlabeled non-specific competitor DNA *hrdB* (N) and specific probe *4189p* (S). Arrows: free probes. Bracket: SAV4189-DNA complex.

To determine whether SAV4189 directly regulates *aveR* and *sav_4189*-*sav_4190* operon, we performed EMSAs using soluble His_6_-SAV4189 protein expressed in *E. coli*. The *aveR* and *sav_4189* promoter regions were labeled as probes *aveRp* and *4189p*, respectively (**Figure 3A**). Probe *hrdB* and BSA were used as negative probe and protein controls. His_6_-SAV4189 did not bind to probe *aveRp* or *hrdB*, but bound specifically to probe *4189p* and generated several clearly retarded bands (**Figure 3B**), suggesting that SAV4189 binds more than two sites on its own promoter region. Binding specificity was confirmed by competition experiments using ∼100-fold excesses of unlabeled specific probe *4189p* (lane S), which reduced binding of SAV4189 to labeled probe *4189p*, and of non-specific probe *hrdB* (lane N), which did not reduce the retarded signals. BSA did not retard probe *4189p*, even at high protein concentration (750 nM). These findings indicate that the positive regulatory effect of SAV4189 on *aveR* expression is indirect, and that SAV4189 directly regulates *sav_4189*-*sav_4190* operon through binding to the *sav_4189* promoter region.

### Determination of Precise SAV4189-Binding Sites

**FIGURE 4:**
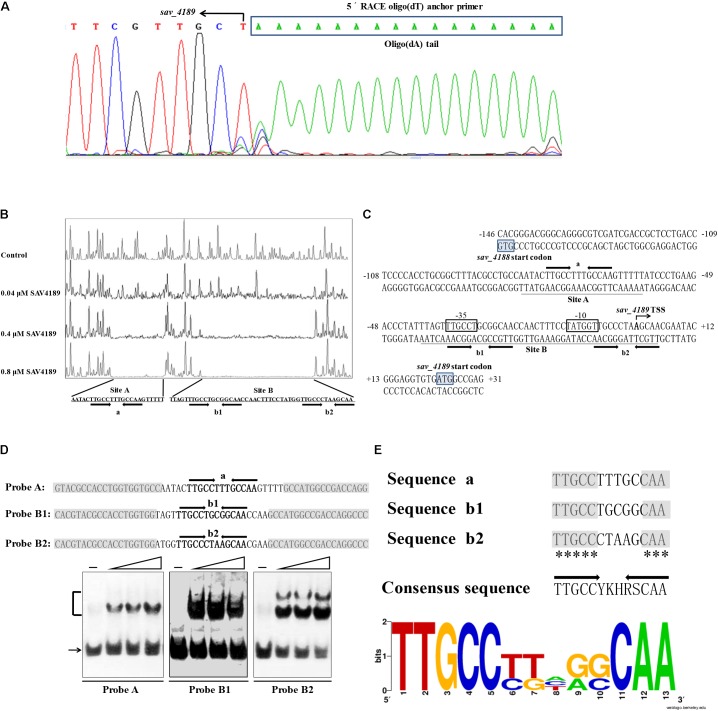
*sav_4189* promoter structure and SAV4189-binding sites. **(A)** Determination of *sav_4189* TSS by 5′-RACE PCR. Box: complementary sequence of oligo(dT) anchor primer. Arrow: complementary base of TSS. **(B)** DNase I footprinting assay of SAV4189 on its own promoter region. Top fluorogram: reaction with 10 μM BSA (control). Protection patterns were acquired with increasing His_6_-SAV4189 concentrations. **(C)** Nucleotide sequences of *sav_4189* promoter region and SAV4189-binding sites. Numbers: distance (nt) from *sav_4189* TSS. Shading: translational start codons. Bent arrow: *sav_4189* TSS and transcription orientation. Boxes: potential –10 and –35 regions. Underlining: SAV4189-binding sites. Straight arrows: inverted repeats. **(D)** EMSAs of His_6_-SAV4189 with probes A, B1, and B2. Each lane contained 0.3 nM labeled probe. Each probe was 59 bp. Non-specific DNA sequences from *hrdB* ORF (shaded) were fused with intact 13-bp palindromic sequences a, b1, and b2 (boldfaced) to generate probes A, B1, and B2, respectively. Lanes –: EMSAs without His_6_-SAV4189. Lanes 2, 3, and 4: 62.5, 125, and 250 nM His_6_-4189. **(E)** Consensus sequence analysis of SAV4189-binding sites by WebLogo program. Asterisks: consensus bases. Arrows: inverted repeats. Height of each letter is proportional to appearance frequency of corresponding base.

To identify precise binding sites and elucidate the regulatory mechanism of SAV4189 on its target promoter, we performed 5′ RACE and DNase I footprinting assays. The *sav_4189* TSS was localized by 5′ RACE to an A residue at position 22 nt upstream of the *sav_4189* translational start codon (**Figures 4A,C**), leading to the probable -10 and -35 promoter regions shown by boxes in **Figure 4C**.

DNase I footprinting assay with a 511-bp FAM-labeled probe covering the *sav_4188*-*sav_4189* intergenic region revealed that SAV4189 protected two sites (termed A and B) on the *sav_4189* coding strand (**Figure 4B**). The two sites are 17 nt apart. Site A extends from positions -82 to -59, and site B extends from positions -41 to +5, relative to the *sav_4189* TSS (**Figure 4C**). Site A is close to, and site B overlaps the putative -35 and -10 regions of *sav_4189* promoter, indicating that SAV4189 represses transcription of *sav_4189*-*sav_4190* operon by hindering recruitment of RNA polymerase.

MarR-family transcriptional regulators generally form homodimers and bind palindromic sequences within target promoter regions (Perera and Grove, 2010; Grove, 2013). DNAMAN analysis of the SAV4189-binding sites revealed that site A contained one palindromic sequence (termed “a”), and site B contained two such sequences (termed “b1” and “b2”) (**Figures 4B,C**). The three palindromic sequences were all 13 bp (5-nt inverted repeats separated by 3 nt) and highly similar. To assess contributions of the three 13-bp sequences to SAV4189 binding, we performed EMSAs using 59-bp probes containing intact 13-bp sequences; i.e., both sides of sequences a, b1 and b2 were fused with non-specific DNA fragments from *hrdB* ORF by PCR to create probes A, B1 and B2, respectively (**Figure 4D**). SAV4189 bound to each of these probes, indicating that all three 13-bp sequences are important for SAV4189 binding, and that SAV4189 binds at the three palindromic sequences on its own promoter region. These findings are consistent with that of several retarded bands in EMSAs for interaction of probe *4189p* with purified His_6_-SAV4189, as described in the preceding section.

WebLogo program^1^ analysis of these three palindromic sequences generated a 13-bp consensus SAV4189-binding sequence 5′-TTGCCYKHRSCAA-3′ (Y = T/C; K = T/G; H = A/C/T; R = A/G; S = C/G) (**Figure 4E**).

### Affinity of SAV4189 Binding to Different Target Sequences

**FIGURE 5:**
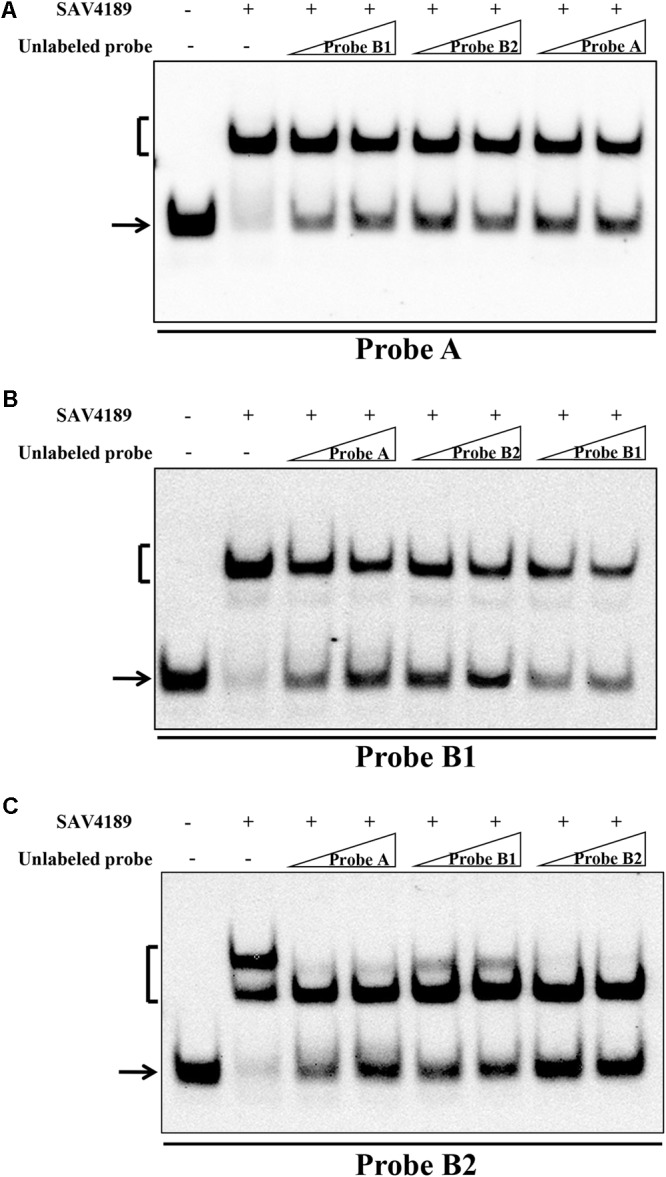
Comparison of the relative affinities of SAV4189 for different target sequences. **(A)** EMSA of His_6_-SAV4189 with labeled probe A and unlabeled probes (A, B1, B2). **(B)** EMSA of His_6_-SAV4189 with labeled probe B1 and unlabeled probes. **(C)** EMSA of His_6_-SAV4189 with labeled probe B2 and unlabeled probes. 0.15 nM labeled probe and ∼50- and 150-fold amounts of unlabeled specific competitor probe were used in competition assays. SAV4189 concentration: 125 nM. Arrows: free labeled probes. Brackets: SAV4189-DNA complexes.

To compare affinity of SAV4189 for its three target sequences, we performed competitive EMSAs using labeled probes A, B1 and B2 that contained sequences a, b1 and b2, respectively, and ∼50-fold and 150-fold excesses of unlabeled specific competitor DNAs. In experiments using 50-fold excesses, unlabeled probe B1 dissociated less SAV4189 from labeled probe A than the other two unlabeled probes (**Figure 5A**). Further analysis using labeled probe B1 (**Figure 5B**) confirmed that affinity of SAV4189 to probe B1 was the lowest among the three probes. In experiments using 150-fold excesses, the dissociation of SAV4189 from labeled probe B2 caused by unlabeled probe B2 was more than that of the other two unlabeled probes (**Figure 5C**), indicating the highest affinity of SAV4189 for probe B2, which was confirmed by comparison of intensities of SAV4189-probe B2 complex corresponding to 50-fold excess of unlabeled probes (**Figure 5C**). The competitive ability of 150-fold excess of unlabeled probe A was higher than that of the same excess of unlabeled probe B1 (**Figures 5B,C**). Therefore, we determined the affinity of SAV4189 to its target sequences in the order b2 > a > b1; however, the differences are slight.

### Prediction of SAV4189 Regulon and Verification of New SAV4189 Target Genes

To decipher SAV4189 regulon in *S. avermitilis*, we used the 13 bp SAV4189 consensus binding sequence to scan the genome with the PREDetector software program^2^. We identified 250 putative SAV4189 target genes using cut-off score ≥ 7. Of these genes, 115 are unknown or unclassified (Supplementary Table S2), and the remaining 135 were assigned to 17 functional groups based on the KEGG pathway database for *S. avermitilis*^3^. 49 of these 135 genes are associated with regulatory functions. These findings reflect the degree of biological significance of SAV4189 in *S. avermitilis*.

**FIGURE 6:**
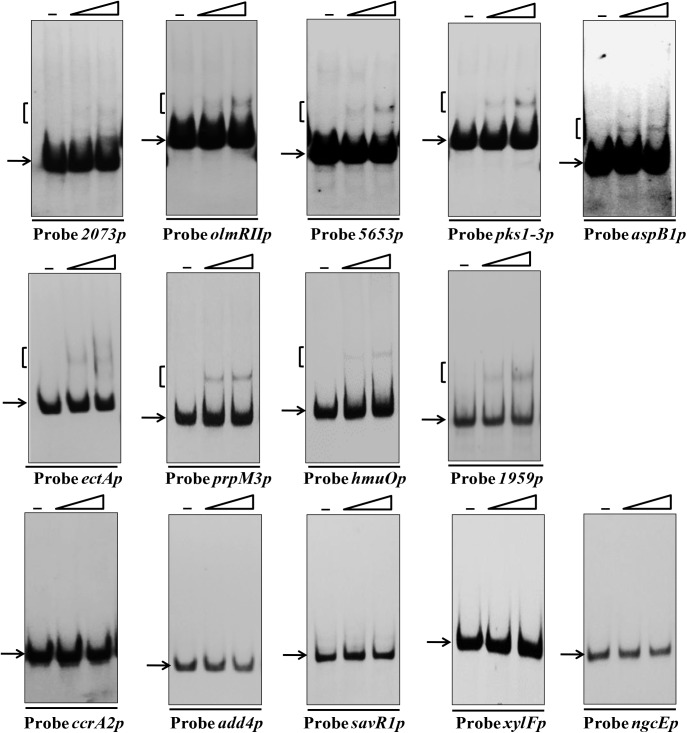
Confirmation of new SAV4189 target genes. EMSAs of His_6_-SAV4189 with promoter regions of 14 predicted target genes. Each lane contained 0.15 nM labeled probe. Lanes –, EMSAs without His_6_-SAV4189. Lanes 2–3 contained 200 and 400 nM His_6_-SAV4189, respectively.

To test the accuracy of bioinformatic analysis, 14 putative target genes from various groups that have relatively high score or are well annotated were selected and confirmed experimentally by EMSAs. Among these, 9 targets were confirmed to bind directly to SAV4189 (**Figure 6**). The newly identified SAV4189 target genes were *sav_2073* (encoding a putative GntR-family transcriptional regulator), *olmRII* (*sav_2901*, encoding a LuxR-family transcriptional regulator), *sav_5653* (encoding a putative ROK-family transcriptional regulator), *pks1-3* (*sav_7362*, encoding a putative modular polyketide synthase), *aspB1* (*sav_2008*, encoding a putative aspartate aminotransferase), *ectA* (*sav_6398*, encoding a L-2,4-diaminobutyrate acetyltransferase), *prpM3* (*sav_3185*, encoding a putative magnesium or manganese-dependent protein phosphatase), *hmuO* (*sav_5930*, encoding a putative heme oxygenase), and *sav_1959* (encoding a putative secreted acyl esterase). SAV4189 did not interact with promoter regions of *ccrA2* (*sav_1911*, encoding a putative crotonyl-CoA reductase), *add4* (*sav_4906*, encoding a putative adenosine deaminase), *savR1* (*sav_3309*, encoding a putative Mrr restriction system protein), *xylF* (*sav_2247*, encoding a putative simple sugar ABC transporter substrate-binding protein), and *ngcE* (*sav_2251*, encoding a putative ABC transporter substrate-binding protein).

### *sav_4189* Overexpression and *sav_4190* Deletion Enhance Avermectin Yield in Industrial Strain A-144

**FIGURE 7:**
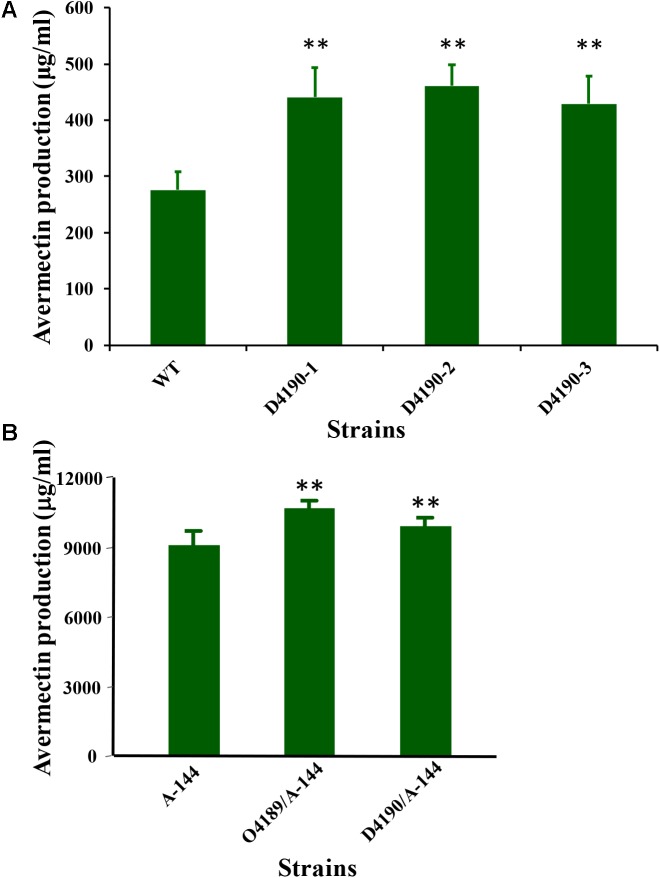
Avermectin yield in various *S. avermitilis* strains grown in FM-I for 10 days. **(A)** WT and *sav_4190* deletion mutant strains D4190-1, -2, and -3. **(B)** Industrial strain A-144 and its derivatives (O4189/A-144: *sav_4189* overexpression strain; D4190/A-144: *sav_4190* deletion strain). ^∗∗^*P*< 0.01.

The findings that SAV4189 positively regulates avermectin production, and that *sav_4190* is a target gene of SAV4189, suggest that *sav_4190* has a functional role in avermectin production. To test this concept, we constructed *sav_4190* deletion mutant D4190 (Supplementary Figure S1B). Avermectin yields were ∼56–67% higher in strains D4190-1, -2, and -3 than in WT (**Figure 7A**), indicating that *sav_4190* plays a negative role in avermectin production. *sav_4190* expression level on day 2 for D4189 grown in FM-I was ∼24-fold higher than that of WT (**Figure 2B**), indicating that altered *sav_4190* expression contributes to the reduced avermectin yield of D4189.

Because avermectin yield was strikingly increased by *sav_4189* overexpression and by *sav_4190* deletion, we experimentally engineered these two genes in industrial strain A-144 and evaluated effects on avermectin yield. *sav_4189* overexpression vector pKC1139-4189 and *sav_4190* deletion vector pD4189 were introduced into A-144 to construct mutants O4189/A-144 and D4190/A-144, respectively. Avermectin yield in shake-flask fermentation was ∼21% higher for O4189/A-144 and ∼15% higher for D4190/A-144 relative to parental A-144 (**Figure 7B**). *sav_4189* overexpression and *sav_4190* deletion thus appear to be effective strategies for further enhancement of avermectin production in industrial strains.

### *sav_4189* Overexpression in *S. coelicolor* Promotes Antibiotic Production

**FIGURE 8:**
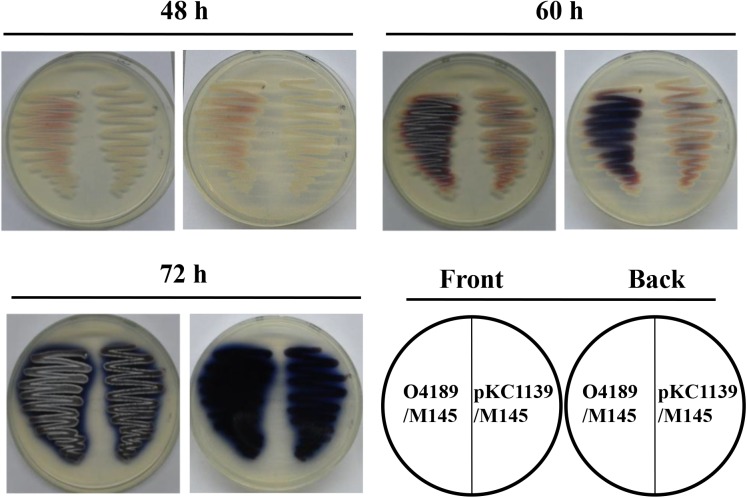
Effect of *sav_4189* overexpression on antibiotic production in *S. coelicolor* M145. *S. coelicolor* strains were grown on YBP agar at 28°C. O4189/M145: *sav_4189* overexpression strain of M145. pKC1139/M145: M145 carrying control plasmid pKC1139.

SAV4189 homologs are distributed widely in *Streptomyces*. To investigate possible regulation of antibiotic production by SAV4189 in other *Streptomyces* species, we transformed *sav_4189* overexpression vector pKC1139-4189 into model strain *S. coelicolor* M145. Resulting transformant O4189/M145 was grown on YBP agar, and earlier, higher antibiotic production was visually observed (**Figure 8**). This finding suggests that SAV4189 (or its homolog) acts as an activator of antibiotic production in *Streptomyces* species other than *S. avermitilis*. Thus, a strategy for strain improvement based on engineering of SAV4189 and its target gene may be extended to other industrial *Streptomyces* strains having SAV4189 homologs, to promote antibiotic production.

### HygB and Thi Act as Ligands of SAV4189

**FIGURE 9:**
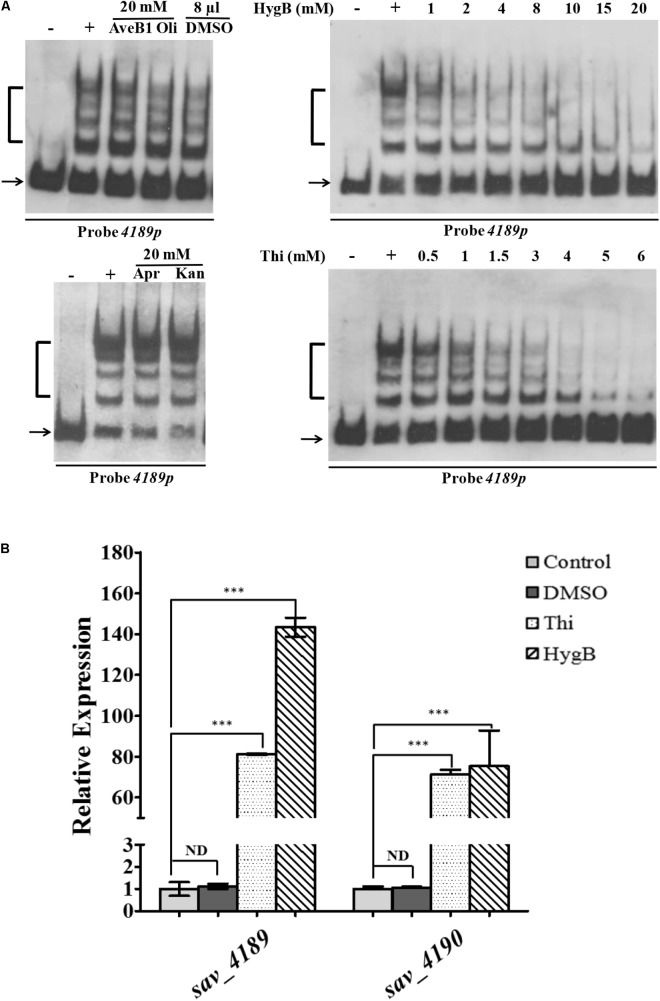
Effects of ligands on SAV4189 DNA-binding activity. **(A)** EMSAs of His_6_-SAV4189 with various antibiotics. 125 nM His_6_-SAV4189 was used for Apr and Kan; 62.5 nM His_6_-SAV4189 was used for other antibiotics. DMSO was used as solvent control. **(B)** Effects of HygB (10 μg/ml) and Thi (diluted in DMSO; final concentration 5 μg/ml) on transcription of SAV4189 target genes *in vivo*. Control: no antibiotic or DMSO added. DMSO: DMSO (but no antibiotic) added. Value of each gene was shown as fold change relative to control value, defined as 1. NS, not significant; ^∗∗∗^*P*< 0.001.

A defining characteristic of MFRs is their response to specific ligands, such as antibiotics and phenolic compounds, resulting in attenuated DNA-binding activity and derepressed gene expression (Perera and Grove, 2010; Grove, 2013). In view of the finding that SAV4189 regulates antibiotic production, we performed EMSAs to evaluate effects of various antibiotics on SAV4189 affinity for probe *4189p*. SAV4189 DNA-binding activity was not inhibited by endogenous antibiotics Oli and AveB1, nor by exogenous antibiotics Kan and Apr, even at high concentration (20 mM) (**Figure 9A**). In contrast, SAV4189-*4189p* complex was disrupted in dose-dependent manner by exogenous HygB and Thi (**Figure 9A**), suggesting that SAV4189 may sense and respond to these antibiotics to reduce repression of its target genes. This concept was tested by measuring expression of SAV4189 targets *sav_4189* and *sav_4190* in WT in response to exogenous HygB and Thi. Cells were cultured in FM-II to stationary phase (96 h), added with these antibiotics at subinhibitory concentration, and total RNAs were isolated from fermentation culture after 3 h and analyzed by qRT-PCR. *sav_4189* and *sav_4190* transcription levels were notably increased by addition of HygB or Thi, but not of DMSO (**Figure 9B**). HygB caused ∼143-fold increase of *sav_4189* and ∼75-fold increase of *sav_4190* expression. Thi caused ∼81-fold increase of *sav_4189* and ∼71-fold increase of *sav_4190* expression. These findings, taken together, indicate that SAV4189 recognizes various exogenous antibiotics such as HygB and Thi as ligands to modulate its DNA-binding activity and transcription of target genes.

## Discussion

In this study, we characterized a previously unknown MFR, SAV4189, as a strong activator of avermectin biosynthesis, and identified the SAV4189 regulon in *S. avermitilis*. Of the 42 MFRs of *S. avermitilis*, SAV4189 is the first to be characterized. The present findings enhance our understanding of control of secondary metabolism by MFRs in *Streptomyces*. Results of qRT-PCR and EMSAs indicate that SAV4189 plays an indirect role in stimulation of avermectin production by altering transcription of the CSR gene *aveR*, whose product is essential for activating all *ave* structural genes (Guo et al., 2010). The observation that SAV4189 indirectly activates *aveR* transcription suggests a possibility that it controls *aveR* through a yet-unknown cascade mechanism. However, the candidate SAV4189 targets listed in Supplementary Table S2 do not include reported *aveR* upstream regulator genes *phoP* (Yang et al., 2015), *avaR1* (Zhu et al., 2017), or *avaR2* (Zhu et al., 2016). Future studies will lead to identification of direct regulator(s) of *aveR* that mediate SAV4189 activation, and clarify the molecular mechanism underlying SAV4189 function in avermectin production.

We identified two SAV4189 target genes: *sav_4189* and its adjacent cotranscribed gene *sav_4190*. Overexpression of *sav_4189* in both WT and industrial strains strongly promoted avermectin production, indicating that endogenous SAV4189 is not at saturated level. Enhancement of SAV4189 expression is therefore an effective approach for increasing avermectin yield. *sav_4189* is negatively autoregulated, indicating that SAV4189 utilizes this mechanism to strictly regulate its expression level and consequent avermectin production. We proposed that SAV4189 mainly activates avermectin production during early growth of *S. avermitilis* when *sav_4189* transcription reaches a maximum level. As the level of SAV4189 expression increases, the transcription of its own gene is repressed, resulting in low expression level during late stage to avoid overproduction of avermectins. SAV4189 homologs are widespread in *Streptomyces*, and *sav_4189* overxpression in *S. coelicolor* enhances antibiotic production. Therefore, the strategy of increasing yield by engineering of SAV4189 or its homologs (e.g., SCO4032 in *S. coelicolor*) may be extended to other industrially important antibiotics produced by *Streptomyces*.

*sav_4190*, which encodes a transmembrane efflux protein belonging to the major facilitator superfamily (MFS), is repressed directly by SAV4189. *sav_4190* expression level is low in WT strain; however, deletion of this gene results in increased avermectin yield in both WT and industrial strains, suggesting that *sav_4190* plays an important negative role in avermectin production. In O4189, though *sav_4190* transcription was undetectable on day 6, there is still a very low *sav_4190* transcription level on day 2. Thus, deletion of *sav_4190* in *sav_4189* overexpression strain may further improve avermectin yield.

The MFS transport system is widespread in bacteria, archaea, and eukarya, and has great diversity of function. According to Pao et al. (1998), MFS members can be classified into 17 distinct families, each of which transports a single class of structurally related compounds. Getsin et al. (2013) categorized transport proteins in *S. coelicolor* on the basis of their substrates. The putative substrates transported by SCO4031, a SAV4190 homolog in *S. coelicolor*, are multiple drugs. However, the substrates of SAV4190 could not be avermectins. If SAV4190 pumps out avermectins directly, deletion of *sav_4190* will reduce avermectin yield. The fact is that SAV4190 has a negative effect on avermectin production. Thus, SAV4190 may be involved in expulsion from cells of intermediates of the avermectin biosynthetic pathway or some other precursors needed for avermectin biosynthesis when they accumulate to a certain level, thereby avoiding excessive avermectin production. We demonstrated recently that another MFS transporter in *S. avermitilis*, SAV7490 (AveM), also has a strong inhibitory effect on avermectin production, and that *sav_7490* deletion significantly enhances avermectin yields in WT and industrial strains (Liu et al., 2015). Engineering of this type of transport proteins is therefore a promising approach for increasing antibiotic production, and studies are required to elucidate the functions of these proteins in *Streptomyces*.

MarR-family transcriptional regulators are completely conserved in the wHTH DNA-binding domain, but display high variability in the ligand-binding domain, which interacts with a wide variety of ligands (Perera and Grove, 2010). The effective component AveB1 is not the ligand of SAV4189, perhaps because of indirect control of avermectin production by SAV4189. It is not possible at this point to rule out the possibility that avermectin precursors act as SAV4189 ligands. MFR ligands are often related to the regulated genes and the important SAV4189 target *sav_4190* encodes a transmembrane efflux protein which may expel avermectin precursors. It is therefore possible that SAV4190 pumps out SAV4189 ligands during fermentation.

SAV4189 did not bind to endogenous antibiotics AveB1 and Oli, but it did sense exogenous antibiotics HygB and Thi, which are produced by other *Streptomyces* species, to release repression of its target genes. There is increasing evidence for functioning of antibiotics as interspecies signals (Romero et al., 2011; Wang et al., 2014; Zhu et al., 2016). However, only a few receptors for exogenous antibiotic signals have been identified to date. In *S. coelicolor*, pseudo γ-butyrolactone receptor ScbR2 was reported to be the receptor for exogenous antibiotic jadomycin B (JadB), which modulates morphological differentiation and endogenous undecylprodigiosin (Red) production (Wang et al., 2014). We found recently that AvaR2, a ScbR2 homolog in *S. avermitilis*, acts as receptor for exogenous antibiotic signals JadB, HygB, and Apr (Zhu et al., 2016). AvaR2 and ScbR2 are members of the TetR-family transcriptional regulators (TFRs). TcaR from *Staphylococcus epidermidis* is an MFR involved in control of antibiotic resistance. Crystal structures have been determined for complexes of TcaR with the antibiotics ampicillin (Amp), Kan, streptomycin (Strep), methicillin (Meth), penicillin G (PnG), and chloramphenicol (Chl), indicating the remarkable plasticity of the multidrug-binding pocket of this protein (Chang et al., 2010, 2013). The above findings for TcaR, and our observation that SAV4189 responds to various structurally distinct antibiotics, indicate that some MFR proteins also function as receptors for exogenous antibiotic signals. This concept is consistent with the well-known variability of antibiotics produced by different *Streptomyces* species. These species may require differing types of receptors (such as TFRs and MFRs) to sense antibiotic signals produced by neighboring organisms, to elicit appropriate cellular responses. To our knowledge, SAV4189 is the first example of an exogenous antibiotic receptor among *Streptomyces* MFRs, and presumably plays an important role in interspecies communication. Our findings inform further investigations of ecologically relevant interactions between *Streptomyces* MFRs and exogenous antibiotic signals.

The predicted SAV4189 regulon contains 250 genes, which have been extensively cataloged and are involved in numerous physiological processes. EMSA analysis led to identification of 9 new SAV4189 targets. Among these, *sav_2073*, *olmRII*, and *sav_5653* are involved in transcriptional regulation, reflecting the complex cascades of SAV4189 regulatory network. *olmRII* encodes an oligomycin pathway-specific activator (Yu et al., 2012). *pks1-3* encodes a putative modular polyketide synthase that belongs to *pks1* cluster for biosynthesis of an unknown secondary metabolite. These findings indicate a pleiotropic role of SAV4189 in regulation of secondary metabolism. We selected three putative SAV4189 target genes (*aspB1*, *ccrA2*, *add4*) associated with primary metabolism for evaluation and found that *aspB1* involved in aspartate metabolism is SAV4189 target. *ect* genes encode enzymes for biosynthesis of compatible solute ectoine, which plays an important osmoprotective role (Bursy et al., 2008). The finding that SAV4189 binds to probe *ectAp* for *ectA-ectB-ectC-ectD* operon suggests that SAV4189 mediates protective responses to stress signals, such as osmotic stress. The other three identified SAV4189 targets are *prpM3* involved in protein modification, *hmuO* involved in porphyrin metabolism, and *sav_1959* with unknown function. Taken together, the present findings clearly demonstrate the pleiotropic effects of SAV4189 on cell physiological processes in *S. avermitilis*. Further studies in this important species will experimentally confirm the SAV4189 targets, clarify the broader regulatory roles of this protein, and eventually provide a comprehensive picture of cellular responses triggered by antibiotic and stress signals and mediated by SAV4189.

## Author Contributions

YW and XZ designed the research. JG, XZ, XL, and WL performed the experiments. ZC, LD, and JL contributed study materials. YW and JG wrote the manuscript.

## Conflict of Interest Statement

The authors declare that the research was conducted in the absence of any commercial or financial relationships that could be construed as a potential conflict of interest.

## Footnotes

1 http://weblogo.berkeley.edu/

2 http://predetector.hedera22.com/

3 www.genome.jp/kegg-bin/show_organism?org=sma
