# Supplementary material for: SAV4189, a MarR-Family Regulator in Streptomyces avermitilis, Activates Avermectin Biosynthesis
Source: Front Microbiol. 2018 Jun 26;9:1358. doi: 10.3389/fmicb.2018.01358 (PMC6036246; doi:10.3389/fmicb.2018.01358)
Supplement: Supplementary file 1 [file Data_Sheet_1.PDF]

SAV4189, a MarR-family Regulator in *Streptomyces avermitilis*, Activates Avermectin Biosynthesis

Jia Guo[1,2,3], Xuan Zhang[1], Xiaorui Lu[1], Wenshuai Liu[1], Zhi Chen[1], Jilun Li[1], Linhong Deng[2] and Ying Wen[1]*

1. State Key Laboratory of Agrobiotechnology and College of Biological Sciences, China Agricultural University, Beijing, China

2. Institute of Biomedical Engineering and Health Sciences, Changzhou University, Changzhou, China

3. Key Laboratory of Carbohydrate Chemistry and Biotechnology, Ministry of Education, School of Biotechnology, Jiangnan University, Wuxi, China

*** Correspondence:**

Ying Wen

E-mail: wen@cau.edu.cn   Phone: +86-10-62732715

Supplementary Figures:

FIGURE S1: Schematic methods for deletion of *sav_4189* (A) and *sav_4190* (B).

FIGURE S2: Confirmation of transcriptional unit *sav_4189-sav_4190* by RT-PCR.

FIGURE S3: Amino acid sequence alignment of SAV4189 and its homologs in eight *Streptomyces* species.

FIGURE S4: Effects of *sav_4189* deletion and overexpression on morphological differentiation.

Supplementary Tables:

TABLE S1: Primers used in this study.

TABLE S2: Putative targets of SAV4189.

FIGURE S1

A

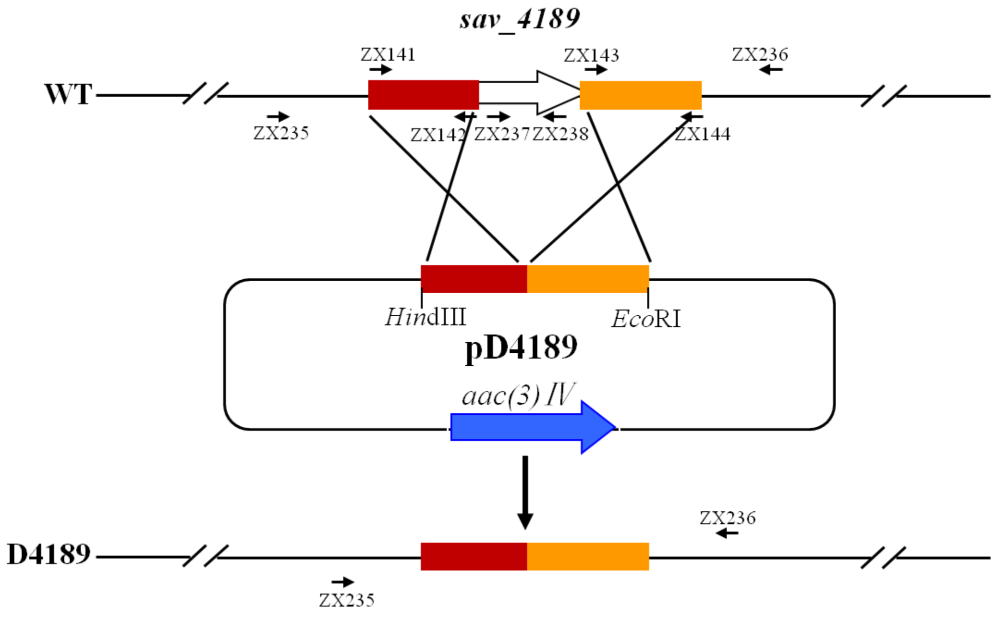

B

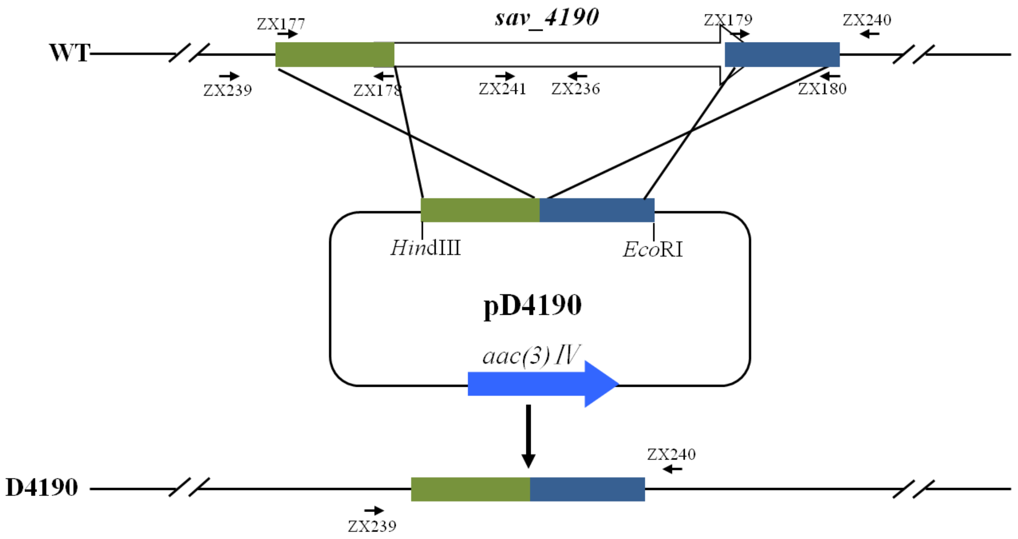

FIGURE S1 | Schematic methods for deletion of *sav_4189* (A) and *sav_4190* (B). Large arrows: genes and their directions. Small arrows: positions of primers used for amplifying exchange regions and confirming gene deletions. Blocks: homologous exchange regions used for gene deletions.

FIGURE S2

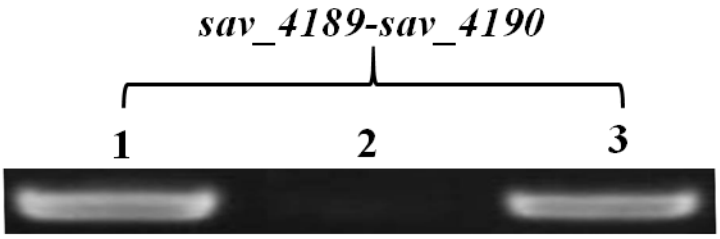

FIGURE S2 | Confirmation of transcriptional unit *sav_4189-sav_4190* by RT-PCR. Lane 1: positive control with WT genomic DNA as template. Lane 2: negative control with WT RNAs (reverse transcriptase omitted) as template. Lane 3: RT-PCR analysis. Primers used for amplifying *sav_4189-sav_4190* intergenic region were ZX226 and ZX227.

FIGURE S3

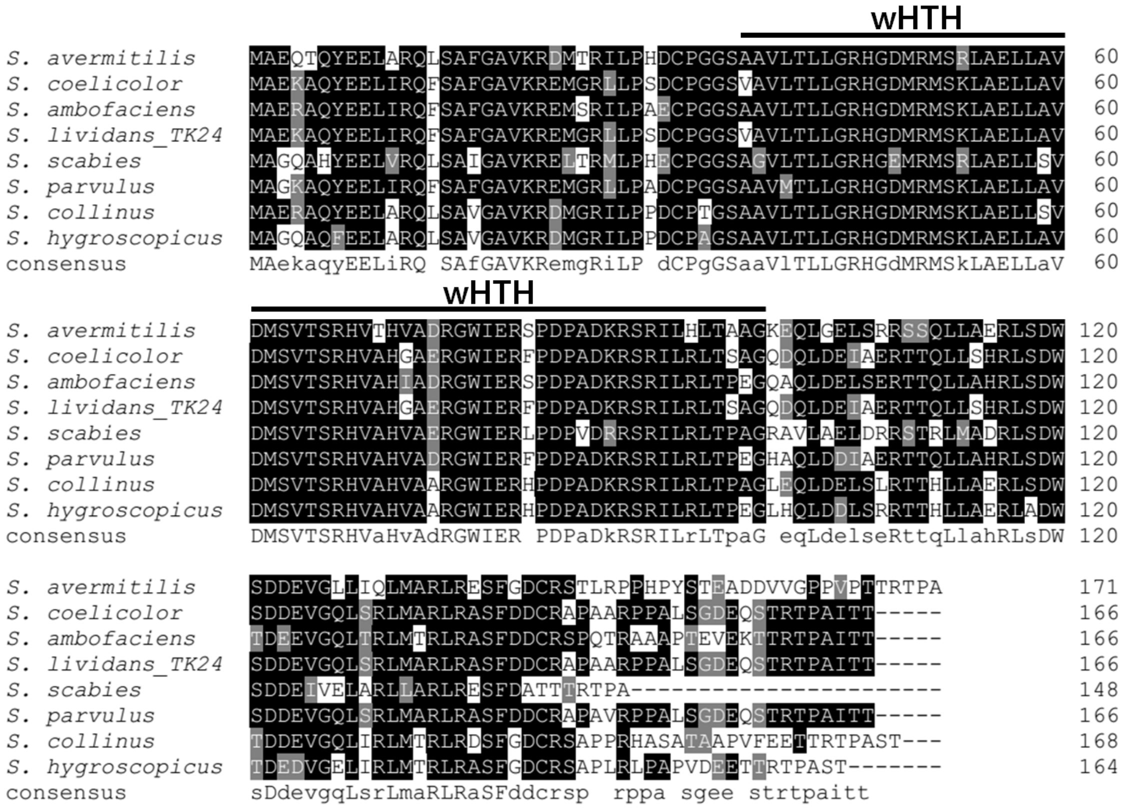

FIGURE S3 | Amino acid sequence alignment of SAV4189 and its homologs in eight *Streptomyces* species. *S. avermitilis* SAV4189 showed high identity with its homologs in the other species, as follows: *S. coelicolor* (77%), *S. ambofaciens* (79%), *S. lividans* (77%), *S. scabies* (72%), *S. parvulus* (77%), *S. collinus* (80%), *S. hygroscopicus* (77%). Black line at top: wHTH domain.

FIGURE S4

A

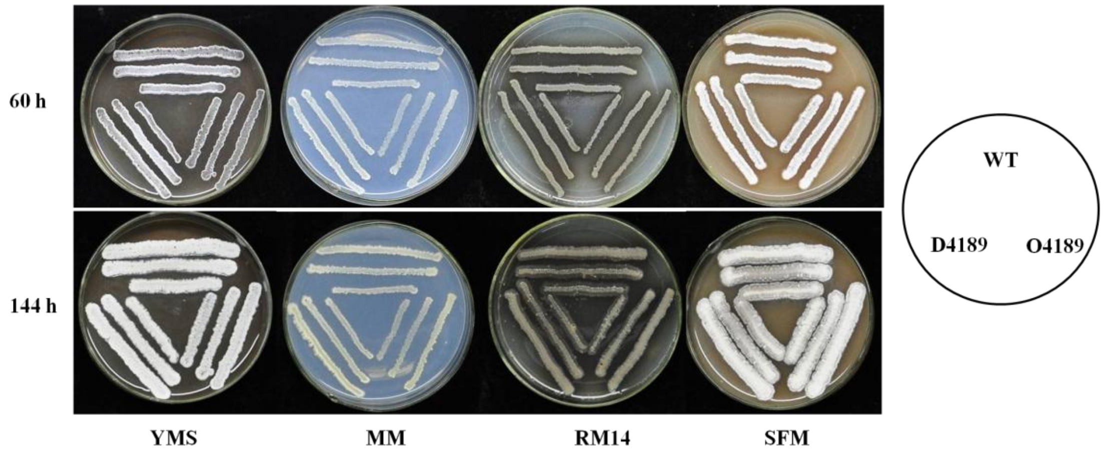

B

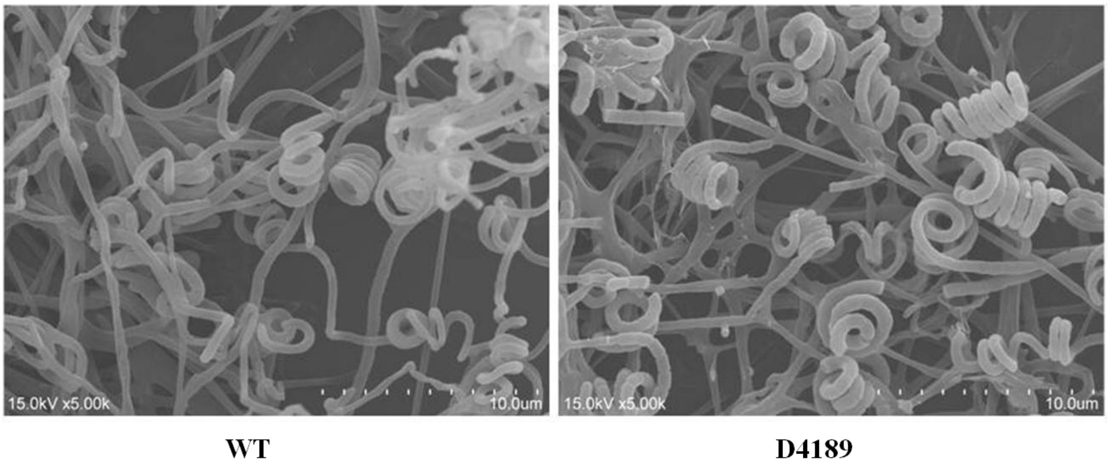

FIGURE S4 | Effects of *sav_4189* deletion and overexpression on morphological differentiation. (A) Phenotypes of WT, *sav_4189* deletion mutant (D4189), and *sav_4189* overexpression strain (O4189) grown on YMS, MM, RM14, and SFM plates at 28 ℃. **(B)** SEM images showing aerial hyphae and spores of WT and D4189 grown on SFM for 4 d.

TABLE S1 | Primers used in this study.

Primer purpose/ primer	DNA sequence (5'-3')	Use
Gene deletion, complementation, and overexpression		
ZX141	CTGAAGCTTACGAAGGCACGGAACATCT, *Hin*dIII	Deletion of *sav_4189*
ZX142	GTGTCTTATGCGGGTGTACGCTGGTCACCGACATGTCCAC	
ZX143	GTGGACATGTCGGTGACCAGCGTACACCCGCATAAGACAC	
ZX144	GCTGAATTCCCGAGGTAGCCGGAGTAAC, *Eco*RI	
ZX235	GACGATGCCGACGATCAG	Confirmation of *sav_4189* deletion in D4189
ZX236	GTAGGTCTGCCACGAGATCC	
ZX237	GACGCAGTACGAGGAACTGG	
ZX238	ATGAGCTGGATGAGCAGACC	
ZX149	ACATGCATGCGCAACGAATACGGGAGGTG, *Sph*I	Complementation of *sav_4189* in D4189 and overexpression of *sav_4189* in *Streptomyces*
ZX150	GCTCTAGAGTGCTTGGCGTGAGAGCC, *Xba*I	
ZX177	CTGAAGCTTGTGAAGCGGGACATGACG, *Hin*dIII	Deletion of *sav_4190*
ZX178	GGTGTACTCGCCGCTGTCGAGAGGGCCTCCATGATCT	
ZX179	AGATCATGGAGGCCCTCTCGACAGCGGCGAGTACACC	
ZX180	GCTGAATTCGGAGATCCCCAGGTGCTG, *Eco*RI	
ZX239	TAAGCAACGAATACGGGAGGT	Confirmation of *sav_4190* deletion in D4190
ZX240	ATAGGTGATCTGCGGGTACG	
ZX241	TCCAGATAGCCCTCGTCATC	
ZX236	GTAGGTCTGCCACGAGATCC	
ZX205	AACCATATGGCCGAGCAGACGCAG, *Nde*I	Overexpression of His_6-SAV4189 in *E. coli*
ZX206	GCTGAATTCCGTGAGAGCCCCGCACAC, *Eco*RI	
EMSAs		
YQ1	GCCTGGTCCTCCGAACTCC	Probe *aveRp*
YQ2	GGTGCAGACAGGAAACTCCCT	
ZX73	CGACCGCTCCTGACCTCC	Probe *4189p*
ZX74	CATCACACCTCCCGTATTCGT	
GJ91	CCAAGGGCTACAAGTTCTCC	Probe *hrdB*
GJ92	TTGATGACCTCGACCATGTG	
ZX63	GTACGCCACCTGGTGGTGCCAATACTTGCCTTT GCCAAGTTTTGCCATGGCCGACCAGG	Probe A
ZX64	CCTGGTCGGCCATGGCAAAACTTGGCAAAGG CAAGTATTGGCACCACCAGGTGGCGTAC	
ZX61	CACGTACGCCACCTGGTGGTAGTTTGCCTGCGGC AACCAAGCCATGGCCGACCAGGCCC	Probe B1
ZX62	GGGCCTGGTCGGCCATGGCTTGGTTGCCGCAGGC AAACTACCACCAGGTGGCGTACGTG	
ZX65	CACGTACGCCACCTGGTGGATGGTTGCCCTA	Probe B2

	AGCAACGAAGCCATGGCCGACCAGGCCC	
ZX66	GGGCCTGGTCGGCCATGGCTTCGTTGCTTA	
	GGGCAACCATCCACCAGGTGGCGTACGTG	
ZX243	TCGGTCAGCCTTGCGTAG	Probe *2073p*
ZX244	TCCGGACTTGACTGATCT	
ZX245	AATTGCTGGCCGAAGCCC	Probe *olmRIIp*
ZX246	CGAGGACCACACCGCCTT	
ZX247	GACGATCTCACCCGCACC	Probe *5653p*
ZX248	CCTTCCGTTCATGCACAC	
ZX249	GTCCTAAACGAGCCATCTACC	Probe *pks1-3p*
ZX250	GCATCGGATTCCTGTGTG	
ZX251	CGGACGATCAGGAGGGTC	Probe *aspB1p*
ZX252	GACTGCCGGAACTCCATG	
ZX253	TGACCCCTCCCGCCACTTC	Probe *ectAp*
ZX254	GCGTCGATAACCCCCATTTTGTC	
ZX255	CGAAGGACTGACGTTTTC	Probe *add4p*
ZX256	GTACGTGCTCCATTCCAG	
ZX257	TGTGGTTCGAGCTGGCGG	Probe *prpM3p*
ZX258	ACATACTCCCAGTCTTCTA	
ZX259	GATCAACGTTGCCAAGGC	Probe *savR1p*
ZX260	GACGACTCATATGCGGCA	
ZX261	ACGGACAGCGGGGCGGACAT	Probe *hmuOp*
ZX262	TCATGGGATCTCCGGTGC	
ZX263	GATGTCGTCGAACTCGTC	Probe *1959p*
ZX264	TCTTGCGTGCGTGTCCCA	
ZX265	GTCAACTCTCGGTGCTTC	Probe *xylFp*
ZX266	CGTGTTCACTTCAGAAAC	
ZX267	CGTGCGTTCGATGTTGCT	Probe *ngcEp*
ZX268	AAGTCCTCGCCTTCTCCA	
ZX269	TGCCATACGTTGAATATGT	Probe *ccrA2p*
ZX270	CGTCCAGGATTTCCTTCAC	
DNase I footprinting assay		
FAM-89footS	ACGAAGGCACGGAACATCT	*sav_4189* promoter region
89footAS	CTGGTCACCGACATGTCCAC	
qRT-PCR		
GJ97	CAGAAGAACTCACGCTCGTC	*aveR* ORF
GJ98	ACTCTTTCCACAGCCCATTC	
ZX9	GAATACGGGAGGTGTGATGG	*sav_4189* ORF
ZX10	CGTCATGTCCCGCTTCAC	
ZX11	GGATCTCGTGGCAGACCTAC	*sav_4190* ORF
ZX12	ATGGAGGAGAGGGTGATGGT	
RT-PCR		
ZX226	AGGTCGGTCTGCTCATCCAG	Amplification of
ZX227	GCCGATCCCAGCACATAGAT	*sav_4189-sav_4190*

		intergenic region
5' RACE analysis		
4189SP1	GGCGAGCAGCTCGGCGAG	Determination of *sav_4189* TSS
4189SP2	CCTGCTCATCCGCATGTC	
4189SP3	GAGCAGCGTGAGCACGGC	
Oligo (dT) anchor primer	GACCACGCGTATCGATGTCGACTTTTTTTTTTTTTTTTV	
Anchor primer	GACCACGCGTATCGATGTCGAC	

TABLE S2 | Putative targets of SAV4189.

#	Accession number	Gene	Function	Score
**Regulatory function (49)**				
1	*SAV_82*		putative TetR-family transcriptional regulator	7.7
2	*SAV_86*		putative erythropoiesis-stimulating protein	8.6
3	*SAV_146*		putative TetR-family transcriptional regulator	7.6
4	*SAV_151*		putative TetR-family transcriptional regulator	8.2
5	*SAV_431*		putative TetR-family transcriptional regulator	7
6	*SAV_458*	*hspR18_1*	putative hsp18 transcriptional regulator	7.1
7	*SAV_520*		putative transcriptional regulator	10.3
8	*SAV_537*		putative Sir2-family regulator protein	8
9	*SAV_554*		putative LacI-family transcriptional regulator	7.3
10	*SAV_566*		putative TetR-family transcriptional regulator	8.3
11	*SAV_663*	*sig6*	putative RNA polymerase ECF-subfamily sigma factor	7.3
12	*SAV_729*		putative LysR-family transcriptional regulator	7.4
13	*SAV_1087*	*sig14*	putative RNA polymerase sigma factor	7.6
14	*SAV_1091*		putative anti-sigma factor antagonist	7.1
15	*SAV_1252*	*cvnA1*	putative sensor-like histidine kinase	7.4
16	*SAV_1362*		putative transcriptional regulator	7.6
17	*SAV_1612*		putative two-component system response regulator	9.3
18	*SAV_1644*		putative regulatory protein	7.8
19	*SAV_1984*		putative GntR-family transcriptional regulator	7.1
20	*SAV_2007*		putative transcriptional regulator	9.6
21	*SAV_2073*		putative GntR-family transcriptional regulator	9.8
22	*SAV_2301*		putative regulatory protein	7
23	*SAV_2652*		putative regulatory protein	8
24	*SAV_2689*		putative MerR-family transcriptional regulator	8
25	*SAV_2901*	*olmRII*	LuxR-family transcriptional regulator	8.4
26	*SAV_2914*		putative transcriptional regulator	7
27	*SAV_3053*	*catR*	putative hydrogen peroxide sensitive repressor	7.3
28	*SAV_3481*		putative two-component system response regulator	7.1
29	*SAV_3631*		putative two-component system sensor kinase	7.4
30	*SAV_3632*		putative regulatory protein	7.4
31	*SAV_3970*		putative CarD-like transcriptional regulator	7.6
32	*SAV_4130*	*bldC*	putative MerR-family transcriptional regulator	7.1
33	*SAV_4189*		putative MarR-family transcriptional regulator	13.9
34	*SAV_4328*		putative regulatory protein	8.4
35	*SAV_4361*		putative AsnC-family transcriptional regulator	7.2
36	*SAV_4735*	*bds*	putative RNA polymerase ECF-subfamily sigma factor (BldN)	7.4
37	*SAV_4758*		putative transcriptional regulator	7.6
38	*SAV_5006*	*pkn19*	putative serine/threonine protein kinase	8
39	*SAV_5013*		putative DNA-binding protein	9.9

40	*SAV_5014*		putative regulatory protein	9.9
41	*SAV_5068*		putative two-component system response regulator	7.4
42	*SAV_5220*		putative GntR-family transcriptional regulator	7.4
43	*SAV_5333*		putative TetR-family transcriptional regulator	7.3
44	*SAV_5653*		putative ROK-family transcriptional regulator	11.1
45	*SAV_5974*		putative LysR-family transcriptional regulator	8.2
46	*SAV_6131*		putative DNA-binding protein	8
47	*SAV_6889*		putative sensor-like histidine kinase	8.8
48	*SAV_6954*		putative IclR-family transcriptional regulator	7.6
49	*SAV_7270*		putative LacI-family transcriptional regulator	8.2
**Secondary metabolism (1)**				
50	*SAV_7362*	*pks1-3*	putative modular polyketide synthase	7.3
**Metabolism of amino acids and related molecules (6)**				
51	*SAV_2008*	*aspB1*	putative aspartate aminotransferase	9.6
52	*SAV_2111*	*mmuM*	putative homocysteine S-methyltransferase	7
53	*SAV_4243*	*pheA*	putative prephenate dehydratase	7.8
54	*SAV_4244*	*serS1*	putative seryl-tRNA synthetase	8.4
55	*SAV_5075*	*gdhA1*	putative NAD-specific glutamate dehydrogenase	7.1
56	*SAV_5451*	*valS*	putative valyl-tRNA synthetase	9.1
**Carbohydrate metabolism (15)**				
57	*SAV_887*		putative glycosyl hydrolase, secreted	8.3
58	*SAV_967*		putative glycosyl hydrolase, secreted	8.5
59	*SAV_1307*	*dexA*	putative oligo glucosidase	7.6
60	*SAV_1641*	*agaB3*	putative alpha-galactosidase	7.8
61	*SAV_1918*	*glxK*	putative glycerate kinase	7.6
62	*SAV_2024*	*gip*	putative hydroxypyruvate isomerase	7.1
63	*SAV_2258*	*acnA*	putative aconitase	7.4
64	*SAV_3533*	*eno*	putative enolase	7
65	*SAV_4362*	*bkdA*	putative 3-methyl-2-oxobutanoate dehydrogenase	7.2
66	*SAV_5359*		putative glycosyltransferase	7.6
67	*SAV_6398*	*ectA*	diaminobutyrate acetyltransferase	9.3
68	*SAV_6953*	*adhA10*	putative alcohol dehydrogenase	9.4
69	*SAV_7181*	*xylB1*	xylulose kinase	7.1
70	*SAV_7182*	*xylA*	xylose isomerase	7.1
71	*SAV_7442*	*lamA2*	putative secreted glucanase	7.9
**Fatty acid and lipid metabolism (5)**				
72	*SAV_524*	*cdh*	putative CDP-diacylglycerol phosphotidylhydrolase	7.1
73	*SAV_665*	*fabG1*	putative 3-oxoacyl-ACP reductase	7.6
74	*SAV_1831*	*fabH2*	putative 3-oxoacyl-ACP synthase III	7.1
75	*SAV_1911*	*ccrA2*	putative crotonyl-CoA reductase	8
76	*SAV_7008*	*fabG8*	putative 3-oxoacyl-ACP reductase	7.3
**Nucleotide metabolism (3)**				
77	*SAV_404*	*pntA*	putative pyridine nucleotide transhydrogenase alpha subunit	7.3

78	*SAV_4906*	*add4*	putative adenosine deaminase	9.1
79	*SAV_6662*	*mtnK*	putative 5-methylthioribose kinase	7.6
**Protein synthesis, folding, and modification (10)**				
80	*SAV_1000*	*sprC*	putative streptogrisin C (secreted serine protease)	8.4
81	*SAV_1128*	*zmp2*	putative griselysin (secreted neutral zinc metalloprotease)	7.4
82	*SAV_1642*	*ptpA2*	putative conventional protein tyrosine phosphatase	7.8
83	*SAV_2764*		putative metallopeptidase, secreted	8.8
84	*SAV_3185*	*prpM3*	putative magnesium or manganese-dependent protein phosphatase	9.7
85	*SAV_3931*	*groEL2*	putative class I heat-shock protein	7.1
86	*SAV_4229*	*prpB7*	putative magnesium or manganese-dependent protein phosphatase	7.2
87	*SAV_4329*	*ppiA*	putative peptidyl-prolyl cis-trans isomerase	8.4
88	*SAV_5411*	*pepG*	putative aminopeptidase G	7.5
89	*SAV_6682*	*prcA*	putative 20S proteasome alpha-subunit	7.4
**DNA synthesis, repair, recombination, modification, and packaging (5)**				
90	*SAV_2928*		putative DNA repair helicase	7.6
91	*SAV_3309*	*savR1*	putative Mrr restriction system protein	9.5
92	*SAV_3728*	*int8*	putative tyrosine-family recombinase/integrase	7.4
93	*SAV_5202*		putative ATP-dependent helicase	9.5
94	*SAV_6833*	*ruvC*	putative Holliday junction nuclease	8.2
**RNA synthesis and modification (1)**				
95	*SAV_2475*	*miaA*	putative delta(2)-isopentenylpyrophosphate tRNA-adenosine transferase	8.2
**Membrane bioenergetics (1)**				
96	*SAV_589*		putative F420-dependent dehydrogenase	7.3
**Metabolism of cofactors and vitamins (4)**				
97	*SAV_412*	*pteD*	cytochrome P450 hydroxylase	7.1
98	*SAV_3517*	*pobA*	putative p-hydroxybenzoate hydroxylase	8
99	*SAV_5930*	*hmuO*	putative heme oxygenase	10
100	*SAV_7469*	*cyp28*	cytochrome P450 hydroxylase	7.3
**Detoxification and adaptation to atypical conditions (8)**				
101	*SAV_459*	*hsp18_1*	putative heat shock protein	7.1
102	*SAV_565*	*bphC*	putative dihydroxybiphenyl dioxygenase	8.3
103	*SAV_1959*		putative acyl esterase, secreted	10.1
104	*SAV_3052*	*katA1*	putative catalase	7.3
105	*SAV_3675*	*pacB1*	putative penicillin acylase, secreted	7.5
106	*SAV_3932*	*cspB2*	putative cold shock protein	7.4
107	*SAV_3947*	*terD2*	putative tellurium resistance protein	7
108	*SAV_4697*	*clpC2*	putative ATP-dependent Clp protease	7.1
**Cell wall and cell envelope (1)**				
109	*SAV_5913*	*dacF*	putative D-alanyl-D-alanine carboxypeptidase	8.9
**Cell division and differentiation (1)**				
110	*SAV_4664*	*tilS*	putative tRNA(Ile)-lysidine synthase	7.5

**Transport and binding proteins (19)**				
111	*SAV_145*		putative membrane transport protein	7.6
112	*SAV_610*		putative MFS transporter protein	8
113	*SAV_891*		putative quaternary amine transporter	7.1
114	*SAV_1508*	*pbuX1*	putative xanthine/uracil permease	7.4
115	*SAV_1558*	*ssuC2*	putative ABC transporter permease protein	8.6
116	*SAV_1560*	*ssuA2*	putative ABC transporter substrate-binding protein	7.4
117	*SAV_1828*	*rbsA1*	putative simple sugar ABC transporter ATP-binding protein	9.1
118	*SAV_2066*		putative xanthine/uracil permease	7.3
119	*SAV_2247*	*xylF*	putative simple sugar ABC transporter substrate-binding protein	7.2
120	*SAV_2251*	*ngcE*	putative ABC transporter substrate-binding protein	9
121	*SAV_3370*		putative simple sugar ABC transporter substrate-binding protein	9.2
122	*SAV_3482*		putative di-tripeptide transporter	7.6
123	*SAV_3676*		putative sodium/proton antiporter	7.5
124	*SAV_5175*		putative osmoprotectant transporter	7.9
125	*SAV_5912*		putative ABC transporter permease protein	8.9
126	*SAV_6276*		putative integral membrane export protein	7.2
127	*SAV_6665*	*glpF1*	putative glycerol uptake facilitator protein	8.2
128	*SAV_7200*	*glpF3*	putative glycerol uptake facilitator protein	7.4
129	*SAV_7271*		putative multiple sugar ABC transporter substrate-binding protein	8.2
**Gas vesicle (1)**				
130	*SAV_599*	*gvpO1*	putative gas vesicle synthesis protein	7.5
**Mobile and extrachromosomal element functions (5)**				
131	*SAV_273*		putative DNA invertase/recombinase	7.2
132	*SAV_309*		putative IS3 family ISVisp1-like transposase	7.3
133	*SAV_3722*		putative IS701 family ISAzvi8-like transposase	8.1
134	*SAV_7545*		putative ISL3 family ISFsp1-like transposase	7.3
135	*SAV_7548*		putative ISL3 family ISFsp1-like transposase	7.3
**Unknown or unclassified genes (115)**				
136	*SAV_14*		putative membrane protein	8
137	*SAV_83*		hypothetical protein	7.7
138	*SAV_87*		hypothetical protein	8.6
139	*SAV_152*		putative dehydrogenase	8.2
140	*SAV_164*		hypothetical protein	7.1
141	*SAV_169*		hypothetical protein	7.4
142	*SAV_304*		hypothetical protein	8.3
143	*SAV_305*		putative membrane protein	8.3
144	*SAV_307*		hypothetical protein	7.1
145	*SAV_362*		putative secreted protein	10.7
146	*SAV_432*		putative secreted protein	7
147	*SAV_493*		putative secreted protein	8.8
148	*SAV_506*		hypothetical protein	7.2

149	*SAV_521*		putative secreted protein	10.3
150	*SAV_536*		hypothetical protein	8
151	*SAV_539*		hypothetical protein	8.2
152	*SAV_553*		hypothetical protein	7.3
153	*SAV_649*	*ard*	putative phosphotransferase	7.3
154	*SAV_662*		hypothetical protein	7.3
155	*SAV_712*		hypothetical protein	7.5
156	*SAV_730*		hypothetical protein	7.4
157	*SAV_756*		putative secreted protein	9.3
158	*SAV_761*		hypothetical protein	9.5
159	*SAV_762*		putative methyltransferase	9.5
160	*SAV_777*		hypothetical protein	7
161	*SAV_866*		hypothetical protein	10
162	*SAV_886*		hypothetical protein	8.3
163	*SAV_892*		hypothetical protein	7.4
164	*SAV_902*		hypothetical protein	7.8
165	*SAV_999*		putative carboxylase-amine ligase	7.8
166	*SAV_1003*		putative dehydrogenase	7.6
167	*SAV_1004*		hypothetical protein	7.6
168	*SAV_1052*		hypothetical protein	8
169	*SAV_1053*		putative sugar hydrolase	8
170	*SAV_1090*		putative secreted protein	7.1
171	*SAV_1160*		hypothetical protein	7.8
172	*SAV_1161*		putative isomerase	7.8
173	*SAV_1220*		hypothetical protein	8.3
174	*SAV_1248*		hypothetical protein	7.6
175	*SAV_1306*		putative methyltransferase	7.6
176	*SAV_1353*		putative secreted protein	8
177	*SAV_1363*		putative secreted protein	7.6
178	*SAV_1557*		hypothetical protein	8.6
179	*SAV_1613*		putative dehydrogenase	9.3
180	*SAV_1806*		putative secreted protein	7.4
181	*SAV_1872*		putative secreted protein	7.1
182	*SAV_1919*		hypothetical protein	7.6
183	*SAV_1921*		putative integral membrane protein	7.6
184	*SAV_1964*		hypothetical protein	8
185	*SAV_1965*		putative secreted protein	8
186	*SAV_1985*		putative oxidoreductase	7.1
187	*SAV_2074*		hypothetical protein	9.8
188	*SAV_2149*		putative copper amine oxidase	7.5
189	*SAV_2252*		putative alpha mannosidase	9
190	*SAV_2426*		hypothetical protein	9.7
191	*SAV_2451*		putative secreted protein	8.7
192	*SAV_2476*		hypothetical protein	8.2

193	*SAV_2653*		hypothetical protein	8
194	*SAV_2688*		hypothetical protein	8
195	*SAV_2934*		hypothetical protein	8.4
196	*SAV_3222*		hypothetical protein	7
197	*SAV_3399*		hypothetical protein	8.9
198	*SAV_3668*		hypothetical protein	7.8
199	*SAV_3901*		hypothetical protein	7.6
200	*SAV_3904*		putative lipoprotein	7.6
201	*SAV_3944*		hypothetical protein	7.2
202	*SAV_3945*	*thiX2*	putative flavin-dependent reductase	7.2
203	*SAV_3946*		hypothetical protein	7
204	*SAV_4080*		putative lipoprotein	10.2
205	*SAV_4097*		hypothetical protein	8.6
206	*SAV_4153*		putative amidase	8.2
207	*SAV_4165*		hypothetical protein	8.2
208	*SAV_4188*		putative membrane protein	13.9
209	*SAV_4228*		hypothetical protein	7.2
210	*SAV_4325*		putative secreted protein	7.8
211	*SAV_4373*		hypothetical protein	8.2
212	*SAV_4392*		putative pirin-like protein	8
213	*SAV_4630*		hypothetical protein	8.2
214	*SAV_4696*		putative proline-rich protein	7.8
215	*SAV_4750*		putative membrane protein	7.1
216	*SAV_4759*		putative dehydrogenase	7.6
217	*SAV_4862*		putative secreted protein	12.6
218	*SAV_4897*		putative peptidase amidohydrolase	10.1
219	*SAV_5011*		putative secreted protein	7.1
220	*SAV_5019*		hypothetical protein	9.5
221	*SAV_5221*		hypothetical protein	7.4
222	*SAV_5266*		hypothetical protein	7.3
223	*SAV_5334*		putative membrane protein	7.3
224	*SAV_5405*		putative secreted protein	7.6
225	*SAV_5412*		hypothetical protein	7.5
226	*SAV_5432*		hypothetical protein	7.3
227	*SAV_5450*		putative membrane protein	9.1
228	*SAV_5591*		putative secreted protein	7.1
229	*SAV_5652*		hypothetical protein	11.1
230	*SAV_5761*		putative protocatechuate dioxygenase	7.1
231	*SAV_5874*		hypothetical protein	7.8
232	*SAV_5875*		putative NGG1-interacting factor	7.8
233	*SAV_5885*		hypothetical protein	7.1
234	*SAV_5973*		hypothetical protein	8.2
235	*SAV_6248*		putative integral membrane protein	7.5
236	*SAV_6622*		hypothetical protein	8.9

237	*SAV_6640*		putative oxidoreductase	7.4
238	*SAV_6641*		putative FMN-dependent monooxygenase	7.4
239	*SAV_6848*		putative secreted protein	8.2
240	*SAV_6857*		hypothetical protein	7.4
241	*SAV_6888*		hypothetical protein	8.8
242	*SAV_6911*		putative ATPase	8.2
243	*SAV_6916*		hypothetical protein	8.2
244	*SAV_6955*	*hcaC2*	putative ferredoxin subunit of phenylpropionate dioxygenase	7.6
245	*SAV_6967*		hypothetical protein	7.4
246	*SAV_6968*		putative MutT-like protein	7.4
247	*SAV_7099*		putative NLP/P60-family secreted protein	9
248	*SAV_7291*		hypothetical protein	7.3
249	*SAV_7298*		putative membrane protein	7.1
250	*SAV_7299*		putative glycine-rich protein	7.1
**Total: 250**				

Yellow highlighting: putative targets selected and confirmed experimentally by EMSAs.
